# Mindfulness possibilities in the treatment of chronic headaches

**DOI:** 10.1192/j.eurpsy.2024.154

**Published:** 2024-08-27

**Authors:** D. Perczel-Forintos, D. Sal

**Affiliations:** Clinical Psychology, Semmelweis University, Budapest, Hungary

## Abstract

**Introduction:**

Headache is a very common health problem worldwide and in our country due to the increasing environmental damage and daily stress. The proportion of patients with headache in general practice is 4-5%, in neurology up to 30%. Chronic headache as a persistent stressor exhausts the body through central sensitisation, which can lead to the consolidation of maladaptive coping strategies such as avoidance, feelings of loss of control, catastrophising pain. This can lead to a deterioration in quality of life and depression also. The effectiveness of pharmacotherapy in coping with chronic pain is limited, so attention should be paid to modifying maladaptive pain behaviour, as recommended by the NICE guidelines. The international literature shows that mindfulness-based cognitive therapy (MBCT) has been shown to be effective in the management of chronic headache, primarily in improving quality of life, increasing self-efficacy and reducing pain catastrophisation and depression (Hunt et al., 2022).

**Objectives:**

Our first objective was to introduce mindfulness-based cognitive therapy in Hungary to patients suffering from chronic headache. Secondly, we wanted to measure the impact of the method on quality of life, coping with pain and depression.

**Methods:**

N=28 patients, suffering from chronic headaches (tension headache and migraine) participated in the study at the Department of Clinical Psychology, Semmelweis University (BNO: G430, G431, G442). Selection criteria were: referral from a neurologist, age 18-65. The intervention was an 8-session mindfulness-based cognitive therapy for pain (Day, 2017) led by an MBCT teacher and a clinical psychology resident. Before the intervention, all patients had an individual first interview and filled in the questonnaires. *Measures:* Beck Depression Questionnaire, Pain Catastrophizing Scale, Comprehensive Headache-related Quality of life Questionnaire, Five Facet Mindfulness Questionnaire, Cognitive Emotion Regulation Questionnaire.

**Results:**

After the intervention, there was a significant reduction in the negative impact of pain on quality of life (p<0.05, Cohen’s d=0.6), pain catastrophization (p<0.01, Cohen’s d=0.74), and depression (p<0.001, Cohen’s d=0.84). In addition, several sub-factors of mindfulness increased, including non-reactivity and being non-judgemental (p<0.05, Cohen’s d=0.57), as well as adaptive cognitive emotion regulation strategies (p<0.05, Cohen’s d=0.49).

**Conclusions:**

We can conclude, that in line with international findings, MBCT has been shown to be effective in reducing the negative impact of depressive symptoms, pain catastrophisation and headache on quality of life, and in helping people to cope with pain more adaptively, primarily through the acquisition of mindfulness skills.

**Disclosure of Interest:**

None Declared

